# Current Guidelines, Common Clinical Pitfalls, and Future Directions for Laboratory Diagnosis of Lyme Disease, United States

**DOI:** 10.3201/eid2207.151694

**Published:** 2016-07

**Authors:** Andrew Moore, Christina Nelson, Claudia Molins, Paul Mead, Martin Schriefer

**Affiliations:** University of Virginia School of Medicine, Charlottesville, Virginia, USA (A. Moore);; Centers for Disease Control and Prevention, Fort Collins, Colorado, USA (C. Nelson, C. Molins, P. Mead, M. Schriefer)

**Keywords:** Lyme disease, Borrelia burgdorferi, bacteria, serologic analysis, PCR, guidelines, laboratory diagnosis, public health, vector-borne infections, United States

## Abstract

In the United States, Lyme disease is caused by *Borrelia burgdorferi* and transmitted to humans by blacklegged ticks. Patients with an erythema migrans lesion and epidemiologic risk can receive a diagnosis without laboratory testing. For all other patients, laboratory testing is necessary to confirm the diagnosis, but proper interpretation depends on symptoms and timing of illness. The recommended laboratory test in the United States is 2-tiered serologic analysis consisting of an enzyme-linked immunoassay or immunofluorescence assay, followed by reflexive immunoblotting. Sensitivity of 2-tiered testing is low (30%–40%) during early infection while the antibody response is developing (window period). For disseminated Lyme disease, sensitivity is 70%–100%. Specificity is high (>95%) during all stages of disease. Use of other diagnostic tests for Lyme disease is limited. We review the rationale behind current US testing guidelines, appropriate use and interpretation of tests, and recent developments in Lyme disease diagnostics.

## Abstract 2

Clinicians must consider patient medical history, timeline of symptoms, and hazards of alternative laboratory tests.

Lyme disease is a tickborne disease caused by spirochetes within the *Borrelia burgdorferi* sensu lato species complex (*1*). In the United States, Lyme disease is caused by *B. burgdorferi* sensu stricto and *B. mayonii* and is transmitted to humans by infected *Ixodes scapularis* or *I. pacificus* ticks (commonly known as blacklegged ticks) (*2*). Lyme disease is the most common vectorborne disease in the United States and causes an estimated 300,000 illnesses annually (*3**,**4*). Cases occur primarily in the northeast and upper midwest regions (Figure 1); however, ecologic and environmental changes have catalyzed a gradual geographic expansion (*5*).

**Figure 1:**
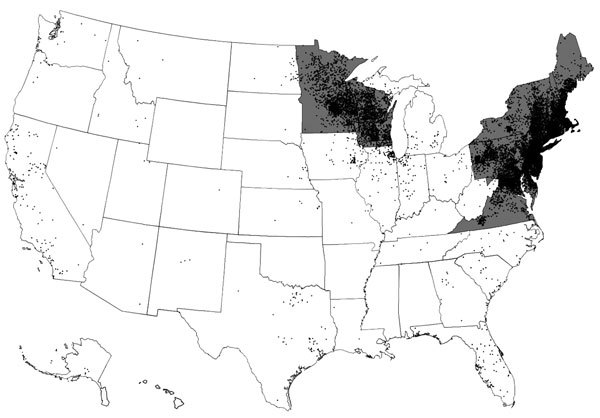
Lyme disease cases (black dots) reported by surveillance, United States, 2005–2010. One dot is placed randomly within the county of residence for each confirmed case. States with the highest incidence of clinician-diagnosed Lyme disease in a large health insurance claims database (gray areas) are also shown. Transmission also occurs in small regions of northern California, Oregon, and Washington. Adapted from (*4*).

There are 3 stages of *B. burgdorferi* infection: early localized, early disseminated, and late disseminated. The classic sign of localized infection is erythema migrans (EM), which is defined as a gradually expanding annular lesion >5 cm in diameter. Approximately 70%–80% of persons with Lyme disease have EM (*1**,**6*). Accompanying signs and symptoms might include fever, lymphadenopathy, myalgias, or arthralgias. If the infection is not treated, the bacteria might spread hematogenously and cause early disseminated Lyme disease, which can manifest as multiple EM skin lesions, facial palsy, meningitis, or carditis. Recurrent large-joint arthritis is the hallmark of late disseminated disease. Late neurologic Lyme disease is uncommon in the United States. Symptoms might include peripheral neuropathy, encephalopathy, or encephalomyelitis.

Patients who have a lesion consistent with EM and live in or have traveled to Lyme-endemic areas can be given a diagnosis without laboratory testing (*6*). In the absence of EM, all other manifestations of Lyme disease require serologic analysis to confirm the diagnosis. The recommended approach for laboratory diagnosis of Lyme disease is a 2-tiered serologic test comprised of an enzyme-linked immunoassay (EIA or ELISA) or immunofluorescence assay (IFA), followed by a reflex Western immunoblot (*7*). When used in accordance with current testing guidelines (*7*), 2-tiered serologic testing is a valuable and highly specific clinical tool for diagnosis of disseminated Lyme disease. Confusion exists, however, among patients and clinicians concerning appropriate use and interpretation of this and other diagnostic tests for Lyme disease (*8**,**9*). In this article, we review the rationale behind current United States testing guidelines, use and interpretation of 2-tiered serologic analysis and other tests in the clinical setting, and recent developments in the field of Lyme disease diagnostics.

## Historical Perspective

The discovery of *B. burgdorferi* as the causative agent of Lyme disease in 1982 prompted development of numerous tests by clinical and private laboratories. Because spirochetes only transiently enter the bloodstream of infected persons in small numbers, direct detection of *B. burgdorferi* by PCR or culture has been challenging (*10*). For this reason, most diagnostic test development has focused on indirect detection of infection by assessing the antibody response of the patient.

**Figure 2:**
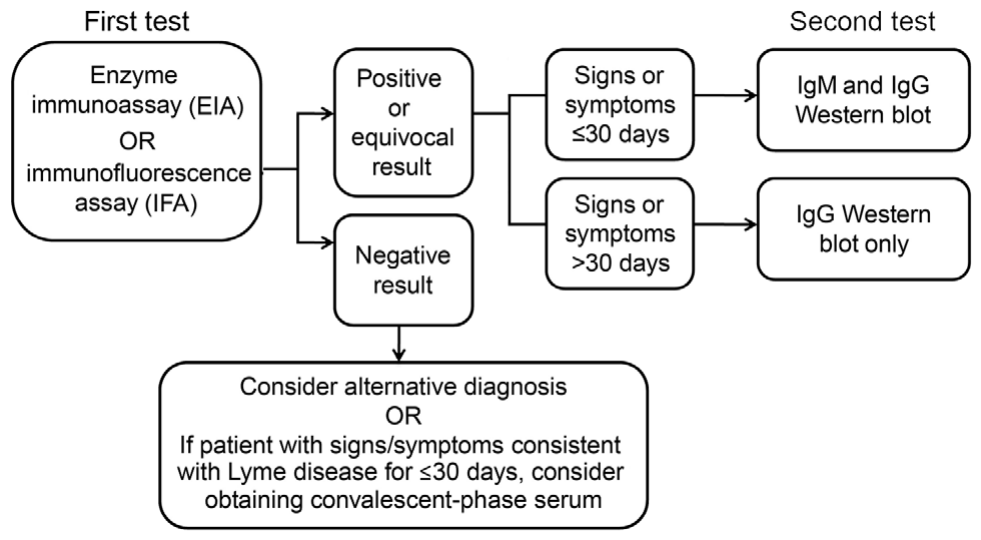
Two-tiered testing for Lyme disease, United States. Adapted from (*7*).

Initially, the variety of serologic tests and lack of concordance among different methods necessitated standardization. In 1994, leading experts convened to review the current evidence and devise a standard testing strategy(*7*). After evaluating the evidence, it became clear that no single test was sufficient on its own. To maximize clinical utility and specificity, the conference diagnostic working group ultimately decided on a 2-tiered serologic testing algorithm (Figure 2). The first tier uses a highly sensitive EIA or IFA that, if the result is positive or equivocal, is followed by a highly specific Western immunoblot as the second-tier test (*7*). Western immunoblot was included in response to a multicenter evaluation of laboratories performing Lyme disease testing, which found that using Western immunoblot in addition to EIA increased specificity to >98%, reducing false-positive results produced by the first-tier EIA (*11*).

## Two-Tiered Serologic Testing

When performed and interpreted in accordance with current guidelines, 2-tiered serologic analysis has a sensitivity of ≈70%–100% and a specificity >95% for disseminated Lyme disease (Table) (*6**,**12**–**15*). Thus, this analysis is the standard of care in diagnosing disseminated Lyme disease but requires appropriate clinical judgment when ordering the test and interpreting the results. To this end, understanding the underlying testing procedure is beneficial.

**Table:** Sensitivity and specificity of serologic tests for patients with Lyme disease, United States*

Variable	Standard 2-tiered algorithm with whole-cell sonicate EIA†			Standard 2-tiered algorithm with C6 EIA,‡ Wormser et al. (*13*)	Two-EIA algorithm§	
	Molins et al. (CDC Lyme Repository) (*14*)	Wormser et al. (*15*)	Branda et al. (*12*)		Branda et al. (*12*)	Wormser et al. (*13**,**15*)
	% Sensitivity (no. tested)					
Early Lyme disease with EM¶						
Acute phase	40 (40)	38 (298)	42 (114)	38 (298)	53 (114)	58 (298)
Convalescent phase	61 (38)	27 (105)	57 (63)#	26 (105)	89 (63)#	67 (105)
Noncutaneous manifestations	96 (46)	94 (142)	87 (55)	93 (142)	100 (55)	97 (144)
Neuritis or carditis	88 (17)	80 (20)	73 (26)	80 (20)	100 (26)	ND
Early Lyme disease with neuritis or carditis	100 (29)	96 (122)	100 (29)	95 (122)	100 (29)	ND
	% Specificity (no. tested)					
Healthy controls						
Endemic area	98 (101)	99 (1,329)	99 (1,146)	99 (1,329)	99 (1,146)	>99 (1,329)**
Nonendemic area	100 (102)	100 (513)	100 (100)	100 (513)	100 (100)	>99 (513)**
Controls with selected other diseases						
Syphilis or RPR positive††	95 (20)	95 (20)	ND	95 (20)	ND	>95 (20)**
Infectious mononucleosis or EBV/CMV positive††	90 (30)	100 (40)	ND	100 (40)	ND	100 (20)
* Helicobacter pylori*	ND	95 (20)	ND	100 (20)	ND	100 (20)
All nonhealthy controls	97 (144)‡‡	99 (366)§§	100 (54)¶¶	100 (366)§§	100 (54)¶¶	100 (366)

**Figure 3:**
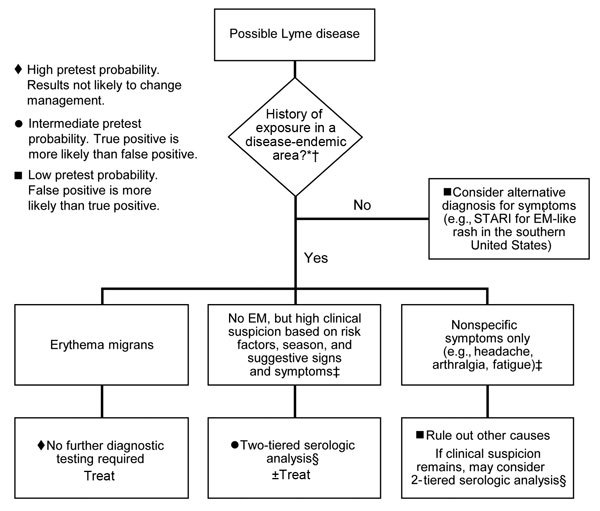
Clinical approach to diagnosis of early Lyme disease, United States. *See Figure 1. †Given the gradual geographic expansion of Lyme disease, testing may be warranted for patients with signs and symptoms of Lyme disease who were exposed in areas that border known disease-endemic regions. ‡For a more detailed discussion of symptoms as they relate to pretest probability, see section on exposure and pretest probability. §For recommended 2-tiered testing protocol, see Figure 2. STARI, Southern tick−associated rash illness; EM, erythema migrans.

*All percentage values were rounded to the nearest whole number. C6, C6 peptide of *Borrelia burgdorferi*; CDC, Centers for Disease Control and Prevention; CMV, cytomegalovirus; EIA, enzyme immunoassay; EM, erythema migrans; EBV, Epstein-Barr virus; ND, not done; RPR, rapid plasma regain. †Standard 2-tiered algorithm: whole-cell sonicate EIA, then IgG (+IgM if presenting within 1 month) Western immune blot if positive or equivocal result. ‡C6+ Western immunoblot algorithm: C6 EIA, then IgG (+IgM if presenting within 1 month) Western immunoblot if positive or equivocal result. §Two-tiered EIA: whole-cell sonicate EIA, then C6 EIA if positive or equivocal result. ¶Patients with EM and epidemiologic risk can be given a diagnosis without serologic analysis (see Figure 3). #Branda et al. (*12*) conducted only convalescent-phase serologic analysis on a well-characterized serum set of Lyme disease patients and controls. All other data points from this study include the data from well-characterized serum set and serum samples submitted to Massachusetts General Hospital (Boston, MA, USA) for routine testing. **Minimum specificity reported by Wormser et al. (*13**,**15*). ††Molins et al. (*14*) tested samples from patients with syphilis or infectious mononucleosis. Wormser et al. (*13**,**15*) tested blood samples with positive results for RPR or CMV/EBV. ‡‡In the report by Molins et al. (*14*), 2-tiered testing had 100% specificity for all other diseases not mentioned above. Other conditions tested include fibromyalgia, severe periodontitis, rheumatoid arthritis, and multiple sclerosis. §§Among patients tested by Wormser et al. (*13**,**15*) there was a single hemolyzed blood sample that showed positive results for all tests. However, both methods of 2-tiered testing had 100% specificity for all other conditions not mentioned above, including *Mycoplasma pneumoniae* infection; HIV; hepatitis A, B, and C; influenza vaccinations; antinuclear antibodies; lipemia; icterus; systemic lupus erythematosus; rheumatoid arthritis; and positive results for rheumatoid factor. ¶¶Includes 25 patients with chronic fatigue syndrome or fibromyalgia, 14 with rheumatic diseases, 9 with neurologic conditions, 5 with infections, and 1 with T-cell lymphoma.

### First Tier

The first-tier test involves measuring the overall antibody response (typically IgM and IgG) of a patient to *B. burgdorferi* antigens. Although both the EIA and IFA have been cleared by the Food and Drug Administration (FDA; Silver Spring, MD, USA) as first-tier tests, laboratories most commonly perform EIA because it is more easily automated. An additional benefit of EIA is that it provides a quantitative value of the relative concentration of antibodies in the serum of a patient compared with that of a control, which enables use of objective cutoff values (*10*).

In the United States, most laboratories use a whole-cell sonicate preparation of *B. burgdorferi* as antigen for the EIA. This test approach has high sensitivity because of multiple antigens in the whole-cell sonicate preparation. However, because some of these antigens are cross-reactive with antigens from the host or other pathogens, specificity of the EIA alone is not optimal (*10*).

Additional FDA-cleared EIAs that use as few as 1 to several antigens, which results in a higher specificity and similar sensitivity than that for whole-cell sonicate EIAs, have recently become commercially available. The cell surface variable-major protein-like sequence expressed (VlsE) lipoprotein and its sixth invariable region, the C6 peptide, are 2 FDA-cleared EIA antigens that are gaining popularity (*16**,**17*). These *Borrelia* antigens are highly conserved and immunogenic among all Lyme borreliosis species and strains, and cause an early antibody response useful for diagnostic testing (*18*).

### Second Tier

Similar to EIA, the second-tier immunoblot is a serologic test that detects antibodies produced against *B. burgdorferi* (*10*). Unlike EIA, however, the immunoblot detects antibodies against a set of preselected *B. burgdorferi* protein antigens. Antibody reactivity to these antigens (indicated by bands on the Western immunoblot) is considered present if bands are visualized with intensity equal to or greater than a control band (*7*).

The specific Western immunoblot test ordered and its subsequent interpretation is dependent on the time course of illness (Figure 2) (*7*). IgM response appears first and is generally directed at the most immunogenic antigens (*19*). Therefore, IgM Western immunoblot should be performed along with IgG Western immunoblot on a reflex basis for patients with signs and symptoms lasting <30 days (*7*). Some patients may require acute-phase and convalescent-phase serologic analysis because of decreased sensitivity during the first weeks of infection (*7**,**10*).

The IgG response generally follows that of IgM and involves a larger number of antigens. Because most patients have a detectable IgG response beyond 30 days, IgG Western immunoblot as the second-tier test is typically sufficient for diagnosis (*19*). At this stage, IgM Western immunoblot is unnecessary and increases the risk for false-positive results.

A positive IgM Western immunoblot result is indicated by the scored presence of >2 of 3 bands (21–24, 39, and 41 kDa), and a positive IgG result is indicated by the scored presence of >5 of 10 bands (18, 21–24, 28, 30, 39, 41, 45, 58, 66, and 93 kDa) (*7*). The 21–24-kDa band represents OspC, an outer surface protein with variable length and amino acid sequence.

It is imperative to avoid interpreting fewer bands as a positive overall result or evidence of infection because antibodies to several antigens are cross-reactive with non-Borrelial antigens. For example, the 41-kDa band indicates reactive antibody against a *B. burgdorferi* flagellin protein. However, this antibody cross-reacts with other bacterial flagellar proteins and was found in 43% of healthy controls in 1 study, including many persons with little or no exposure risk for Lyme disease (*17*). Therefore, presence of 1 IgM band or <4 IgG bands does not indicate an overall positive result. Overinterpreting a small number of antibody bands leads to reduced specificity and potential misdiagnosis (*9**,**20*).

## Additional Diagnostic Tests

### Antibody Testing of Cerebrospinal Fluid

Testing for intrathecal antibody production is integral in the diagnosis of Lyme neuroborreliosis in Europe, where multiple *Borrelia* species and high background seroprevalence limit the usefulness of serologic analysis (*1*). In the United States, the presence of serum antibodies in the appropriate clinical setting is highly sensitive and specific for Lyme neuroborreliosis, making 2-tiered serologic analysis the diagnostic test of choice in most instances (*6**,**10*). Adjunctive testing for intrathecal antibody production is highly specific and might be helpful in confirming the diagnosis, particularly in regions of high seroprevalence. However, a negative result is insufficient to rule out Lyme neuroborreliosis except in cases of encephalomyelitis.

When testing for intrathecal antibodies, it is essential to note that antibodies in serum are passively transferred to cerebrospinal fluid (CSF) in some patients with Lyme disease (*10**,**21*). To control for this transfer, CSF and serum should be collected on the same day and diluted to match the total protein or IgG concentration. A CSF/serum IgG EIA optical density ratio >1.0 indicates active intrathecal antibody production.

### PCR and Culture

PCR can provide highly specific evidence of *B. burgdorferi* nucleic acid in a variety of samples, including synovial fluid, skin biopsy tissue, blood, and CSF (*10**,**22*). However, its clinical utility is limited by low sensitivity (particularly for blood and CSF samples) and its potential for contamination (*10**,**23*).

Synovial fluid PCR is >75% sensitive for Lyme arthritis and might be useful in conjunction with other synovial fluid analyses to differentiate Lyme arthritis from other arthritides (*10**,**22*). Comparatively, PCR of CSF is substantially less sensitive, which limits its clinical utility. In 1 US study, PCR testing of CSF yielded positive results for only 38% of patients with early neuroborreliosis and was even less sensitive for late neuroborreliosis (*24*).

Studies of PCR on blood have found that its high specificity is outweighed by its lack of clinical sensitivity and potential for contamination (*10**,**22*). Thus, PCR has not been universally standardized or optimized for diagnosis of Lyme disease. Nevertheless, some clinical laboratories offer PCR testing for *Borrelia* spp., and PCR of blood has shown utility in detection of the novel genospecies *B. miyamotoi* and *B. mayonii* (*25*).

Because *B. burgdorferi* is a slow-growing organism, current culturing methods are labor-intensive and have poor sensitivity. Culturing is generally not recommended for purposes other than research or for corroboration of disease acquired in regions previously unrecognized for risk of infection (*10*).

## Clinical Considerations and Common Pitfalls

### Timing of Testing—Window Period

As with all serologic tests, clinicians must consider the timing of a patient’s illness when ordering and interpreting Lyme disease tests (*6*). Serologic analysis has low sensitivity during the first few weeks of infection while the antibody response is still developing (*10*). This period is known as the window period and is common to all serologic tests. Patients with illnesses suspicious for early Lyme disease but lacking typical EM can present a diagnostic dilemma because serologic test results might be negative at this point (*6*). In these cases, treatment can be administered at the discretion of the clinician, but serologic analysis is necessary to confirm the diagnosis (Figures 2,3).

### Background Seropositivity

Background seropositivity is a major consideration when testing for Lyme disease. In a seroepidemiologic study conducted in New York, 5% of study participants were found to have antibodies against *B. burgdorferi* (*26*). Seropositivity can result from previous exposure because IgM and IgG against *B. burgdorferi* can remain for many years after initial infection (which, incidentally, is why serologic testing is not useful as a test of cure) (*26**,**27*). However, in the seroepidemiologic study in New York, 59% of seropositive patients denied a prior diagnosis of Lyme disease (*26*). In such persons, seropositivity might indicate a false-positive result or be due to a prior undiagnosed infection that either resolved spontaneously or was treated incidentally with antimicrobial drugs prescribed for another indication.

### Reinfection

Because of antibody persistence, serologic diagnosis of patients with possible reinfection poses a major dilemma for clinicians (*28*). In cases of suspected reinfection, a detailed history and physical examination, including a thorough skin examination, are essential because most patients will have EM. For patients without EM, serologic analysis is still recommended but results should be interpreted with caution. In these cases, it might be helpful to conduct acute-phase and convalescent-phase serologic analysis to detect an increase in EIA titer or an increase in the number of antibody bands that might indicate active infection (*10**,**28*).

### Exposure and Pretest Probability

When determining whether to test for Lyme disease, clinicians must consider a patient’s pretest probability (Figure 3) (*8*). Even highly specific tests can show false-positive results when performed for patients with low pretest probability.

The most crucial factor governing pretest probability for Lyme disease is exposure history. A recent retrospective cohort study by Lantos et al. reported a positive predictive value for Lyme disease serologic analysis in the Duke University hospital system in North Carolina (a low-incidence state) of only 10% for patients with no history of recent travel to a disease-endemic region (*29*). In addition, only 0.7% of patients without recent travel history who had potential signs of disseminated infection (arthritis, cranial neuropathies, or meningitis) were ultimately given a diagnosis of Lyme disease, which indicated that even clinical signs consistent with Lyme disease have poor predictive value in low-incidence regions. Furthermore, even EM-like lesions—once considered pathognomonic for Lyme disease—can be caused by other conditions, such as Southern tick-associated rash illness, a tickborne illness found primarily in the southeastern United States for which an infectious etiology has not been identified (*30*).

For these reasons, positive results for Lyme serologic analysis provide little diagnostic value for patients in areas to which this disease is not endemic and with no history of recent travel to disease-endemic areas (Figure 1) (*8**,**31*). When assessing whether an area is endemic for Lyme disease, it is essential to note that surveillance guidelines classify cases on the basis of the patient’s permanent residence, rather than location of exposure (National Notifiable Disease Surveillance System, http://wwwn.cdc.gov/nndss/conditions/lyme-disease/). A recent study of Lyme disease in low-incidence states found that 84% of infected patients reported recent travel to high-incidence regions (*31*). Thus, although cases have been reported in all 50 states, this finding does not indicate that Lyme disease is endemic to all states.

In addition to exposure history, patient signs and symptoms provide useful information regarding pretest probability (*6*). Patients with EM who live in or have traveled to Lyme disease–endemic areas can be given a diagnosis without serologic testing. For patients without EM, headache and arthralgias are the most common symptoms of early Lyme disease (*32*). However, such symptoms are nonspecific and do not justify serologic testing unless clinical suspicion is high. Signs such as cranial nerve palsy, meningitis, carditis, and migratory large joint arthritis are more suggestive of Lyme disease and improve pretest probability for patients with epidemiologic risk for Lyme disease (*29*). Such signs in at-risk patients generally justify serologic testing. Conversely, gastrointestinal or upper respiratory symptoms are rarely seen in Lyme disease and suggest an alternative diagnosis (*32*).

### Surveillance versus Clinical Diagnostic Testing

One misconception is that 2-tiered serologic analysis is intended only for surveillance, rather than patient diagnosis. This misconception is an apparent conflation of clinical serologic testing recommendations for Lyme disease and the surveillance case definition of the Council of State and Territorial Epidemiologists (*7*) (http://wwwn.cdc.gov/nndss/conditions/lyme-disease/). Recommendations for 2-tiered testing are meant to aid the diagnosis of individual patients in the clinical setting. Serologic test results might be used by public health officials to determine whether a given illness meets the surveillance case definition, but the methods themselves were not developed for this purpose. Furthermore, for practical reasons, serologic results might be used slightly differently in surveillance than is recommended in the clinical setting. For example, although it is not recommended to perform Western immunoblot without a first-tier EIA for laboratory diagnosis, a positive IgG result by Western immunoblot alone is accepted as laboratory evidence of infection for surveillance purposes (http://wwwn.cdc.gov/nndss/conditions/lyme-disease/). This operational definition enables simplification of reporting practices because it can be difficult to track down records of the first-tier test. However, it does not represent best clinical practice.

### Unvalidated Tests and Interpretation Criteria

Several alternative testing centers use laboratory-developed tests that are not currently subject to FDA regulations and might not be clinically validated (*9**,**33*). Alternative laboratories might also use standard Western immunoblot techniques but apply nonstandard interpretation criteria or fail to perform the recommended first-tier EIA. These laboratories often claim to specialize in testing for tickborne diseases and assert that their tests have better sensitivity than standardized 2-tiered serologic analysis.

False-positive results for alternative tests or unvalidated interpretation criteria can lead to patient confusion and misdiagnosis (*9**,**20**,**33*). A recent evaluation of laboratories by Fallon et al. reported an alarming false-positive rate of 58% for samples from healthy control patients submitted to an alternative testing center that used unvalidated criteria to interpret IgM and IgG immunoblots (*34*). Moreover, evaluation of published results from a laboratory claiming to have a new *Borrelia* culture method demonstrated that results were highly suspicious for laboratory contamination (*33**,**35*). Additional alternative tests, such as urine antigen tests and CD57 tests, have also been shown to be inaccurate (*36**,**37*).

It is recommended that clinicians only use Lyme disease tests that have been clinically validated and cleared by the FDA (*16**,**33*). If there is ever any question regarding testing protocols or interpretation, clinicians should consult an infectious disease specialist.

## Future Directions in Diagnostic Testing

### Novel 2-Tiered Algorithms

A great deal of research has focused recently on improving early diagnosis of Lyme disease and reducing subjectivity inherent in Western immunoblot techniques. When used as a stand-alone test, the C6 EIA is more sensitive than the current 2-tiered test for patients with early Lyme disease (64% vs. 48%) but is hampered by decreased specificity (98.4% vs 99.5%) and thus is more prone to false-positive results (*12**,**17*). To address this issue, Branda et al. proposed a 2-tiered EIA approach consisting of 2 FDA-cleared EIAs: whole-cell sonicate EIA followed by reflex C6 EIA. This approach provided a higher sensitivity for early Lyme disease (61% vs. 48% for 2-tiered testing) and equivalent specificity (99.5%) to the current approach (Table) (*12*). A 2-tiered EIA with VlsE EIA followed by reflex C6 EIA has also been proposed. The ease of automation and straightforward results of 2-EIA approaches make them particularly appealing because they would be easier to perform and eliminate the subjectivity of Western immunoblot. Further research is still needed, but in the future, the 2-tiered EIA approach might prove to be a valid alternative for diagnosis of Lyme disease.

### Additional Novel Diagnostic Approaches

Another approach to improve sensitivity for detection of early Lyme disease involves identifying diagnostic proteins and metabolites in serum of patients with Lyme disease. These methods, referred to as proteomics and metabolomics, respectively, are particularly appealing because they also have the potential to identify biomarkers indicative of cure (*38**,**39*). Researchers have also reported promising results using immuno-PCR, which combines the sensitivity of PCR with EIA-based antibody detection (*40*).

### Lyme Serum Repository for Validation of Novel Diagnostic Tests

When developing new tests or assessing their performance, researchers must have access to well-characterized positive and negative controls. Moreover, it is essential to include samples from patients with diseases that have overlapping clinical features and that are known to be serologically cross-reactive because sensitivity and specificity are heavily dependent on the types of patient samples used. However, collecting and characterizing a wide variety of clinical samples for this purpose can be challenging, costly, and time-consuming.

To improve availability of serum sample sets to evaluate novel diagnostic tests, the Centers for Disease Control and Prevention (Fort Collins, CO, USA) and the National Institutes of Health (Bethesda, MD, USA) have developed a repository of well-characterized serum samples from patients with Lyme disease (*14*). The repository includes samples from patients with various stages of Lyme disease; patients with cross-reactive conditions, such as multiple sclerosis and infectious mononucleosis; and healthy controls from both disease-endemic and non–disease-endemic areas. Panels of serum, along with accompanying clinical and laboratory testing results, are now available to researchers for validation of novel diagnostic testing.

## Conclusions

In the United States, 2-tiered serologic analysis is currently the diagnostic test of choice for all patients with signs of extracutaneous Lyme disease. When considering testing, clinicians must take into account the patient’s history, timeline of symptoms, and pretest probability to accurately order the test and interpret the test result. Moreover, clinicians should understand the hazards of alternative laboratory tests and only use FDA-cleared diagnostic tests. Ongoing and published research promises to improve diagnosis of early Lyme disease and reduce subjectivity of the second-tier Western immunoblotting.
